# Dietary intake of carbohydrates in pregnant women with type 1 diabetes—A narrative review

**DOI:** 10.1002/fsn3.1982

**Published:** 2020-12-12

**Authors:** Ann B. Roskjær, Björg Ásbjörnsdóttir, Inge Tetens, Anni Larnkjær, Christian Mølgaard, Elisabeth R. Mathiesen

**Affiliations:** ^1^ Department of Nutrition, Exercise and Sports University of Copenhagen Denmark; ^2^ Center for Pregnant Women with Diabetes Copenhagen Denmark; ^3^ Department of Endocrinology Rigshospitalet Copenhagen Denmark; ^4^ Institute of Clinical Medicine Faculty of Health Sciences University of Copenhagen Denmark; ^5^ Pediatric Nutrition Unit Rigshospitalet Copenhagen Denmark

**Keywords:** carbohydrate quality, carbohydrate quantity, dietary reference intake, pregnancy

## Abstract

In pregnant women with type 1 diabetes, a low but sufficient, intake of carbohydrates is important to aim for near normal glycemic control. However, knowledge about the carbohydrate intake in this group is limited. To assess the average quantity and quality of carbohydrate intake in pregnant women with type 1diabetes compared to healthy pregnant women and current dietary reference intakes. A narrative literature search was performed in PubMed, Embase, and Cochrane Library and by using a snow‐ball search technique to identify papers published on studies conducted in industrialized countries within the last 20 years. Intakes of carbohydrate were assessed qualitatively in relation to the Dietary Reference Intakes recommended by the American Diabetes Association and quantitatively as mean intake of dietary fiber. Five observational studies including 810 pregnant women with type 1 diabetes and 15 observational studies with a total of 118,246 healthy pregnant women were identified. The mean total carbohydrate intake was within the Acceptable Macronutrient Distribution Range (45%–64% of energy intake) in both groups. In pregnant women with type 1 diabetes, the average total intake was 218 ± 19 g/day, which was 20% (53 g/day) lower than in healthy pregnant women. Mean intake of dietary fiber in women with diabetes was lower than the recommended adequate intake for healthy women. With the limitations of pronounced heterogeneity across the included studies, pregnant women with type 1 diabetes reported a mean total carbohydrate intake, which was lower than in healthy pregnant women but still within the recommended range.

## Graphical abstract

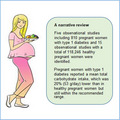

## 1 INTRODUCTION

Glycemic control, with postprandial glycemic excursions within a limited range among pregnant women with diabetes, is of uppermost importance for maternal health and for prevention of adverse pregnancy outcome such as congenital malformations, fetal overgrowth, and preterm delivery (Colstrup et al., 2013; Jensen et al., 2004; Tennant et al., 2015). In addition, avoiding excessive gestational weight gain is important for reducing risk of fetal overgrowth in both healthy (Gaudet et al., 2014) and diabetic pregnancy (Mathiesen, 2016). For pregnant women with type 1 diabetes, strict glycemic control can be obtained mainly through a low, but sufficient, intake of carbohydrates and insulin therapy. The American Diabetes Association (ADA) has set the glycemic target during pregnancy to glycated hemoglobin A1c (HbA1C) below 6% (42mmol/mol) if this can be achieved without significant hypoglycemia (American Diabetes Association, 2020). Both in pregnant women with type 1 diabetes and healthy pregnancies, carbohydrates are essential dietary fuel for growth of the fetus (Hanson et al., 2015). Intake of other macronutrients as protein and lipids as well as micronutrients is also important, but will not be covered in this paper.

The quantity and quality of carbohydrate intake are the main determinants of the postprandial glucose in type 1 diabetes pregnancy (Mathiesen & Vaz, 2008). The ADA recommendations for carbohydrates in women with diabetes follow the recommendations for people with diabetes in general (American Diabetes Association, 2020). In summary, ADA recommends that intakes of carbohydrate to be individually targeted following individual treatment goals and consists mainly of carbohydrates of low glycemic index. The ADA recommendations to pregnant women (American Diabetes Association, 2020) is also based on the dietary reference intake (DRI) given by the National Academy of Medicine (NAM, known as Institute of Medicine (IOM)) with at least 175 g carbohydrate daily. The DRI for all pregnant women of 175 g/day of carbohydrates is recommended regardless of presence of diabetes to cover the glucose utilized by the fetus and brain.

Total carbohydrate intake is recommended to be within the Acceptable Macronutrient Distribution Range (AMDR) of 45% to 65% of total energy intake (2005). However, carbohydrate intake in the upper end of this range makes the blood glucose control more challenging (James et al., 2016). ADA recommends an intake of a minimum of 28 g/day of dietary fiber because the amount of dietary fiber, especially viscous type of fiber, may attenuate the postprandial blood glucose response (Abutair et al., 2016).

Changes in macronutrient distribution and total energy have been seen in the population from the 1970s to the 2010s in national dietary surveys from United States (Austin et al., 2011) and Australia (Grech et al., 2018). In the period 1970s to 2013, national dietary surveys from United States (Austin et al., 2011) have demonstrated an increased consumption of energy and carbohydrates. A similar increase was seen in the Australian population until 1995; thereafter, the intake decreased (Grech et al., 2018). Studies assessing the carbohydrate intake in pregnant women with type 1 diabetes in comparison with healthy pregnant women and recommended intake are limited.

The aim of the present study is to assess the average quantity and quality of carbohydrate intake in pregnant women with type 1 diabetes in relation to healthy pregnant women and current dietary reference intakes.

## 2 METHOD

A narrative review was conducted through a literature search in the following databases: PubMed, Embase, and Cochrane Library and by using a snow‐ball search technique to identify relevant articles. The criteria in selecting the literature were that articles were published in English and within the period 1999–2020.

### 2.1 Search strategy

For the pregnant women with type 1 diabetes, the search terms in Title and abstracts were ‘‘type 1 diabetes’’ AND “pregnancy” AND “carbohydrate” AND “carbohydrate intake”. As the same search terms for healthy women gave no results, the final search in title and abstract was adjusted to “pregnancy” AND “carbohydrate” AND “macronutrient“ AND “dietary intake”.

The literature search was done in the period April 2019 to June 2019.

### 2.2 Selection criteria

The inclusion criteria were as follows: observational cross‐sectional studies; published after January 1998; report of daily carbohydrate intake; singleton pregnancy in women and conducted in western countries with type 1 diabetes or in healthy women. In addition, only papers that describe their use of validated dietary assessment tools, including Food Frequency Questionnaire (FFQ), dietary records or from 24‐hr recall were included. Studies were excluded if the participating women had other health conditions than diabetes that might influence dietary intake. If multiple publications were available and collected from the same data set, the publication that reported that largest number of nutrient variables was selected for inclusion.

### 2.3 Estimation of dietary intake

In studies where information on total carbohydrate, sugar, and dietary fiber intake was provided only in grams, energy intake, or in energy percent, calculations were performed to provide data on intake expressed in energy percent. Simple descriptive statistics with mean carbohydrate intake and standard deviation (*SD*) of the included studies were calculated for diabetic and healthy women, respectively. Due to a very large range in the individual number of participants in each study the different population size was not taken into account. Carbohydrate intake during pregnancy in the background population was reported for early, mid and late pregnancy, separately. The population characteristics and the method of reporting of the carbohydrate intake varied considerably from study to study and further statistics were therefore not applied.

## 3 RESULTS

### 3.1 Carbohydrate intake in pregnant women with type 1 diabetes

**FIGURE 1:**
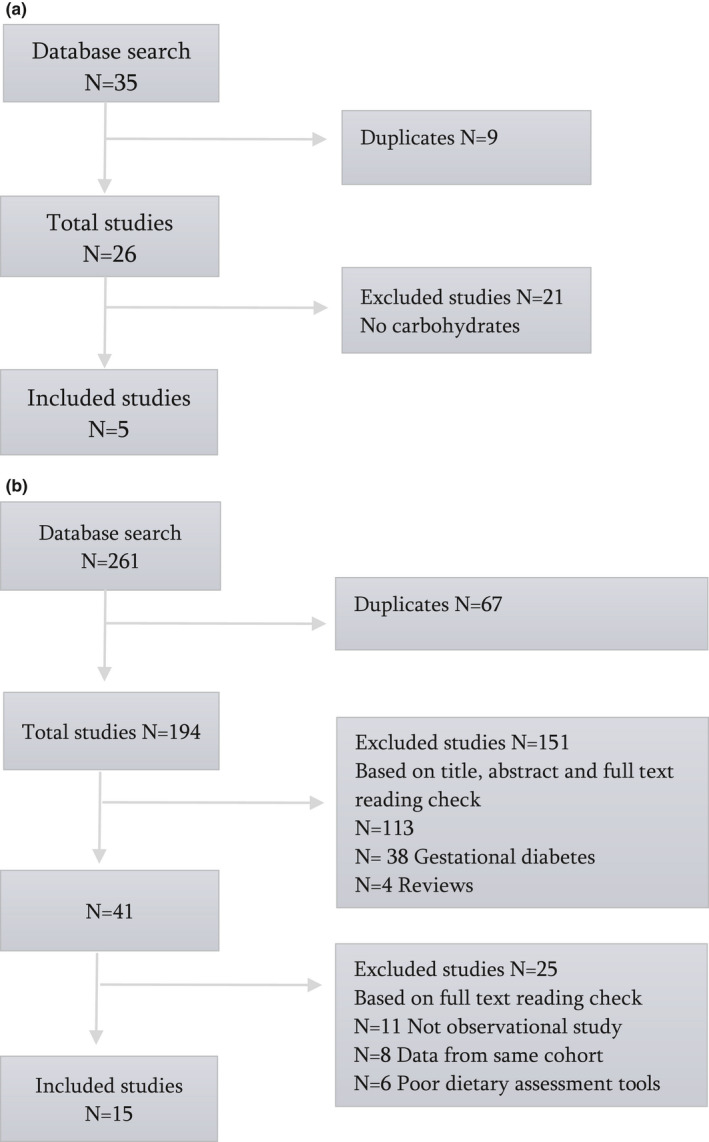
(a) Flow chart of the included studies regarding carbohydrate intake among pregnant women with type 1 diabetes. (b) Flow chart of the included studies regarding carbohydrate intake among healthy 13 pregnant women

A total of 35 articles were identified, where 9 studies were duplicates (Figure 1a). The screening for relevance by title and abstract or full text reading excluded 21 articles, leaving five articles for the narrative review. The five studies were conducted in different countries (Denmark, UK, Canada, and Poland) and they varied in size from 26 to 555 participants (Table 1). The most frequently used dietary assessment method employed was a dietary record (Ásbjörnsdóttir et al., 2017; Kozlowska et al., 2018; Neoh et al., 2018) followed by a Food Frequency Questionnaire (FFQ) (Hill et al., 2012) and a 24‐hr dietary recall (McManus et al., 2013).

**Table 1:** The mean dietary intake of energy, total carbohydrate, sugar, and dietary fiber among pregnant women with type 1 diabetes

Study	Term of pregnancy	No of women	Dietary assessment method	Energy intake (kJ)	Total carbohydrate g/day E%	Sugar g/day	Fiber g/day	
Ásbjörnsdóttir et al. (2017)	Early	107	Dietary record	‐	205^c^	NA	‐	‐
Hill et al. (2012)	Mid	555	FFQ^b^	6,745	221	55	‐	21
McManus et al. (2013)	Mid	29	24‐hr dietary recall	8,058	251	51	‐	20
Kozlowska et al. (2018)	Mid	26	Dietary record	7,088	215	46	27^d^	22
Neoh et al. (2018)	Late	93	Dietary record	7,000	198	50	91^e^	15
Sum/Total mean	‐	810	‐	7,401 ± 498	218 ± 19	51 ± 3	NA	20 ± 3^f^

^a^ Part of a randomized placebo‐controlled trial. Reference: McCance DR, Holmes VA, Maresh MJA et al. Vitamins C and E for prevention of pre‐eclampsia in women with type 1 diabetes (DAPIT): a randomized placebo‐controlled trial. Lancet. 2010;24:259–266.

^b^ Food Frequency Questionnaire.

^c^ Major source of carbohydrates (e.g., bread, potatoes, rice, pasta, dairy products, fruits, and candy) and estimated amount from minor source of carbohydrate (e.g., vegetables, nuts, almonds, meatballs, and sauces) was set to 25 gram carbohydrate.

^d^ Added sugar; ^e^Nonrecommended source of carbohydrates (e.g., sugar, biscuits, cakes, nonalcoholic beverages).

^f^ The average of sugar intake is not calculated due to the small sample size and the heterogeneity in the definition of intake.

In total 810 pregnant women with type 1 diabetes were included, with a mean total dietary carbohydrate intake of 218 ± 19 g/day and 51 ± 3 energy percent (Table 1). Due to different definition of sugar intake and small number of samples, the average intake could not be calculated. However, two studies (Kozlowska et al., 2018; Neoh et al., 2018) describe the amount of sugar and the mean intake was 27 g/day and 91 g/day, respectively. Four studies (Hill et al., 2012; Kozlowska et al., 2018; McManus et al., 2013; Neoh et al., 2018) describe intake of dietary fibers with a mean intake of 20 ± 3 and thereby lower than the recommended intake of 28 g/day by ADA (American Diabetes Association, 2020). The mean energy intake was 7,401 ± 498 kJ per day (Table 1).

### 3.2 Carbohydrate intake during healthy pregnancy

A total of 261 articles were identified, where 67 studies were duplicates (Figure 1b). The screening for relevance by title and abstract or full text reading excluded 151 articles as not relevant, leaving 43 articles for the narrative review. Altogether, 25 studies were excluded because eleven studies were review articles or intervention studies, data from eight studies came from same cohorts, and six studies used nonvalidated dietary assessments tools, leaving 15 articles to be included.

The 15 studies were conducted in different countries (Australian, Denmark, England, Germany, Iceland, Ireland, Norway, Singapore, and USA) and varied in size from 32 to 68,201 participants (Table 2). The most frequently used dietary assessment method employed to obtain the dietary data was a Food Frequency Questionnaire (FFQ). Dietary records and 24‐hr dietary recalls were also used in three of the studies, and one study used both methods.

**Table 2:** The mean dietary intake of energy, total carbohydrate, sugar, and dietary fiber among healthy pregnant women

Study	Term of pregnancy	No of women	Dietary assessment method	Energy intake (kJ)	Total carbohydrate g/day E%		Sugar g/day	Fiber g/day
Olafsdottir et al. (2005)	Early	436	FFQ^a^	8,651	262	51	64^h^	‐
Rad et al. (2011)	Early	32	Dietary record	9,301	281	52	‐	‐
Murrin et al. (2013)	Early	1,004	FFQ^a^	10,661^b^	306	50	140^i^	‐
Derbyshire et al. (2016)	Early	51	Dietary record	8,715^c^	263	53	‐	39^m^
Rifas‐Shiman et al. (2006)	Early	1,543	FFQ^a^	8,565	224	55	‐	20
Diemert et al. (2016)	Early	200	Dietary record	8,314	239	51	103^j^	24
Okubo et al. (2014)	Early	906	FFQ^a^	8,820	286	57	‐	26
Blumfield, et al. (2012)	Early	141	FFQ^a^	7294^d^	182	41	86^k^	39
Veyhe et al. (2012)	Mid	381	FFQ^a^	8,100	219	46	28^i^	25
Haugen et al. 2008)	Mid	40,108	FFQ^a^	9,753^e^	313	54	‐	‐
Siega‐Riz et al. (2002)	Mid	2,247	FFQ^a^	1,184^f^	372	55		24
Shapiro et al. (2016)	Mid	1,079	Dietary record	8,640	251	50	15^h^	18
Chong et al. (2015)	Mid	835	Dietary record	7,962	234	52	‐	‐
Knudsen et al. (2013)	Mid	68,201	FFQ^a^	9,900^g^	319	56	‐	27
Scholl (2004)	Late	1,082	Dietary record	10,058	309	56	49^l^	15
Sum/total mean	‐	118,246	‐	9,105 ± 605	271 ± 47	52 ± 4	NA	± 7^n^

^a^ Food Frequency Questionnaire. ^b,c,d,e,f,g^Calculated cutoff limits to unrealistic reporting of energy intake were used. ^h^Defined as; add sugar. ^i^Defined as; sugar. ^j^Defined as; monosaccharide and saccharose. ^k^Defined as; fructose, glucose, sucrose, maltose, lactose, and galactose. ^l^Defined as; sucrose. ^m^Defined as: southgate fiber and Englyst fiber. ^n^The average of sugar intake is not calculated due to the heterogeneity in the definition of intake.

In total 118,246 healthy pregnant women were included, with a total mean energy intake of 9,105 ± 605 kJ/day, and the amount of carbohydrate intake was 271 ± 47 g/day corresponding to 52 ± 4 energy percent (Table 2). Mean total carbohydrate intakes were above the RDA values in all studies (Derbyshire et al., 2016; Rifas‐Shiman et al., 2006; Diemert et al., 2016; Okubo et al., 2014; Blumfield, et al., 2012; Veyhe et al., 2012; Siega‐Riz et al., 2002; Shapiro et al., 2016; Knudsen et al., 2013; Scholl, 2004).

Data of dietary fiber (Blumfield, et al., 2012; Derbyshire et al., 2016; Diemert et al., 2016; Haugen et al., 2008; Knudsen et al., 2013; Okubo et al., 2014; Rifas‐Shiman et al., 2006; Scholl, 2004; Shapiro et al., 2016; Siega‐Riz et al., 2002; Veyhe et al., 2012) were available in 10 studies with a total mean intake of 26 ± 8 g/day. Separate data for sugar intake were given in seven studies (Blumfield, et al., 2012; Diemert et al., 2016; Murrin et al., 2013; Olafsdottir et al., 2005; Scholl, 2004; Shapiro et al., 2016; Veyhe et al., 2012), but the definition of sugar intake differed considerably and the average intake could not be calculated. In nine of fifteen studies, food frequency questionnaire was used to estimate dietary intake.

The mean carbohydrate intake in pregnant women with type 1 diabetes was on average 53 g/day lower than in healthy women but still well above the minimum requirement of 175 g/day (Table 1). The mean dietary fiber was 20 ± 3 g/day in pregnant women with diabetes and 26 ± 8 g/day in healthy women and both lower than recommended by ADA.

In five of the studies (Blumfield, et al., 2012; Diemert et al., 2016; Okubo et al., 2014; Rad et al., 2011; Siega‐Riz et al., 2002), data on daily intake of energy and carbohydrate intake were collected at least twice during healthy pregnancy (Table 3). The mean total carbohydrate intake and carbohydrate intake presented as energy percent were almost stable during a healthy pregnancy with small changes from minus 3% to plus 8% during pregnancy in the individual five studies.

**Table 3:** Carbohydrate intake in early, mid, and late pregnancy of healthy women

Study	Energy intake (kJ)			Carbohydrate g/day		
	Early	Mid	Late	Early	Mid	Late
Diemert et al. (2016)	8,314	8,653	9,000	239	245	254
Rad et al. (2011)	9,237	9,496	9,525	281	281	281
Siega‐Riz et al. (2002)		8,565	8,941	‐	270	277
Okubo et al. (2014)	8,820	‐	9,586	286	‐	309
Blumfield, et al. (2012)	7,294	‐	7,200	182	‐	179

## 4 DISCUSSION

This review provides a new insight of dietary carbohydrate intake in pregnant women with type 1 diabetes. The mean total carbohydrate intake of was around 20% lower in pregnant women with type 1 diabetes compared with the intake of healthy pregnant women. The total carbohydrate intake was 218 ± 19 g/day and thus above the RDA value of 175 g/day (American Diabetes Association, 2020; Mathiesen & Vaz, 2008), and when expressed as energy percent, the total carbohydrate intake was within the AMDR (45–64 energy percent). Intake of dietary fiber was below the recommended adequate intake of 28 g/day (American Diabetes Association, 2020; Mathiesen & Vaz, 2008).

It is reassuring that pregnant women with type 1 diabetes reported a mean total carbohydrate intake which was lower than in healthy pregnant women but still within the recommended range. This relatively low mean carbohydrate intake probably improves the probability of a lower postprandial increase in plasma glucose fluctuations around meals.

Only one (Blumfield, et al., 2012) out of 15 studies in healthy women reported energy percent of carbohydrates below the range of AMDR. However, in this study, the mean energy intake was reported to be considerably lower (7,294 kJ) compared with the other studies, suggesting that the Food Frequency Questionnaire (FFQ) used in this study may not have been able to full depict an accurate dietary intake.

Considering the long‐term focus on the role of sugar intake in relation to health, it is of note that separate data for sugar intake were only available from two studies of pregnant women with type 1 diabetes. In which even report high heterogeneity in the intake at 27 and 91 g/day, respectively, that may relate to differences in the definitions of added sugar or cultural differences of the cohorts.

Overall, the separate intake for sugar intake was available from less than half of the studies of healthy pregnancy. Patients with type 1 diabetes are recommended to minimize their intake of added sucrose (American Diabetes Association, 2020), and this recommendation is especially underlined for pregnant women with type 1 diabetes, where added sugar particularly should be reduced to a minimum (American Diabetes Association, 2020). In a review of healthy pregnancies, women reported approximately 100 g of sugars intake per day where 50 g came from added sugar (Blumfield et al., 2012).

The dietary fiber was lower than recommended by ADA in both pregnant women with diabetes and in healthy pregnancy and lowest among the women with diabetes. Studies reporting the glycemic index of the carbohydrate intake in pregnant women with type 1 diabetes were not identified.

In total, the quality of carbohydrates intake was not documented to be superior among pregnant women with type 1 diabetes when compared to healthy women. Whether the intake of simple sugars for episodes of hypoglycemia may account for this remains speculative. Both the amount and quality of carbohydrates are important for the diabetic women to know in order to tailor the insulin dose to the carbohydrate intake.

Mean carbohydrate intake was reported several times in pregnancy in five of the studies in healthy pregnancies (Blumfield, et al., 2012; Diemert et al., 2016; Okubo et al., 2014; Rad et al., 2011; Siega‐Riz et al., 2002). The mean total carbohydrate intake and carbohydrate intake expressed, as energy percent, was almost stable during healthy pregnancy. This is in accordance with a systematic review (Blumfield, et al., 2012) reporting that the percentage of energy provided by carbohydrate was stable during pregnancy in industrialized countries. Extra energy is often reported required during pregnancy for growth and maintenance of the fetus, placenta, and maternal tissues (2005). However, energy requirements during pregnancy are complex and influenced by many factors, including physical activity. Thus, energy homeostasis in late pregnancy may be achieved by a reduction in physical activity without extra energy intake (Meltzer et al., 2008; Renault et al., 2012). Cumulative reductions in basal metabolic rate during pregnancy are also reported (Forsum & Lof, 2007).

### 4.1 Strengths and weaknesses

This review is based on a strict search strategy and a comprehensive search in both PubMed, Embase, and Cochrane Library. To our knowledge, this is the first review summarizing the total carbohydrate intake in pregnant women with type 1 diabetes. However, the number of studies identified in this group was low. At the same time, the number of studies in healthy pregnant women was relatively high, improving the external validity for this group. To improve the assessment of carbohydrate intake, data were expressed both as a total daily carbohydrate intake g/day and as the energy percent of total energy intake. However, the study has also several limitations. First, the low number of studies identified for the pregnant women with type 1 diabetes and the pronounced heterogeneity across all studies regarding study design, study duration, eligibility criteria, and cohort sizes. The different dietary methods are of a special concern, as this increases the potential for response bias and measurement error. However, it is acknowledged that some of the differences between the studies may be due to the different dietary assessments tools (Prentice et al., 2011). In general, estimates of dietary intake obtained from Food Frequency Questionnaires (FFQs) tend to be somewhat higher compared to estimates obtained from dietary records (Thomson, 2003), probably due to a lower number of food items, estimated rather than weighed portion sizes and other factors. This is important to keep in mind when comparing different estimates of dietary intake obtained from different dietary assessments tools.

However, as we took a critical appraisal of the dietary assessment methods and only included studies with validated food frequency questionnaire, dietary records, and 24‐hr recall methods, the major sources of potential bias have been excluded.

## 5 CONCLUSION AND PERSPECTIVES

The mean total carbohydrate intake in pregnant women with type 1 diabetes was lower than in healthy pregnant women but still within the recommended range. Mean dietary fiber intake was lower than recommended for pregnant women regardless of presence of diabetes, and focus on sufficient fiber intake is needed. However, it is a limitation that there was a pronounced heterogeneity across all studies regarding study design, eligibility criteria, and cohort sizes. Additional studies of total carbohydrate intake including data on fiber and sugar intake with their relation to glycemic control and pregnancy outcome are needed in pregnant women with type 1 diabetes before more stringent recommendations for dietary carbohydrate intake during pregnancy can be developed. Focus on the minimum carbohydrate intake that is safe to recommend in pregnant women with diabetes is urgently needed.

## 6 TRANSPARENCY DECLARATION

The lead author affirms that this manuscript is an honest, accurate, and transparent account of the study being reported. The lead author affirms that no important aspects of the study have been omitted and that any discrepancies from the study as planned have been explained.

## CONFLICT OF INTEREST

None of the authors had any conflict of interest.
